# Perinatal Care in Canada

**DOI:** 10.1186/1472-6874-4-S1-S28

**Published:** 2004-08-25

**Authors:** Beverley Chalmers, Shi Wu Wen

**Affiliations:** 1 Sunnybrook and Women's College Health Science Centre, University of Toronto, 790 Bay Street, Toronto, Canada; 2 Health Canada, Ottawa, Canada

## Abstract

**Health Issue:**

Canada's standard of perinatal care ranks among the highest in the world, but there is still room for improvement, both in terms of regional differences in care and global comparisons of approaches to care in Canada and elsewhere. Data from the Canadian Perinatal Surveillance System (CPSS) was used to evaluate morbidity and mortality among mothers and infants.

**Key Findings:**

Maternal mortality rates in Canada dropped to 4.4 per 100,000 live births in 1993–1997 and are among the lowest in the world. Rates of Caesarean section increased from 15.3 per 100 deliveries in 1994 to 19.1 in 1997. Although the infant mortality rate in Canada is among the lowest in the world (5.3–8.8 per 1,000 live births 1990–2000), there are unacceptable disparities between subpopulations. In Aboriginal populations, rates of stillbirth and perinatal mortality are 2–2.5 times the Canadian average. There has been a steady increase in the proportion of births among older women who have the highest risk of preterm births and pregnancy complications.

The increasing rate of multiple births has accelerated recently and is of concern as these carry a higher risk of complications and are associated with an increased risk of preterm birth. The costs to the health care system are likely to be high.

**Data Gaps and Recommendations:**

CPSS data, including economic indicators, needs to be collected in a more timely and uniform manner across Canada. The CPSS should provide an evaluation of how well Canada fares in relation to international standards of perinatal care.

## Background

Canada is fortunate to be numbered among the leading countries of the world in terms of its standards of perinatal care. Both infant and maternal mortality rates, key indicators of perinatal health, reflect high standards of care. Despite this, there is still much room for improvement in Canada, both from the point of view of regional differences within the country regarding aspects of perinatal care and in terms of global comparisons of Canadian approaches to perinatal care and those advocated in other regions. This chapter is concerned with ongoing surveillance of Canadian perinatal care with particular reference to abortion, teenage pregnancy, pregnancy in older women and multiple births.

## Methods

A detailed report of Canadian perinatal health indicators is available periodically from the Canadian Perinatal Surveillance System (CPSS) of Health Canada . The findings given in the Results section are based primarily on the 2000 *Canadian Perinatal Health Report*,[1] which provides Canadian figures up to 1997. An updated CPSS report on current perinatal health in Canada is in press at the time of writing.

The CPSS currently monitors 24 perinatal health indicators. These cover both determinants of maternal, fetal, and infant health and perinatal outcomes. Determinants of health that are assessed are behaviours during pregnancy, such as smoking and alcohol intake; education, maternal age and breast-feeding; and health services, such as induction, Caesarean section, operative deliveries, perineal trauma and early discharge practices. Maternal outcomes evaluated include mortality, induced abortion, ectopic pregnancy, severe morbidity and readmission after discharge. Fetal and infant health outcomes measured include preterm births, weight for gestational age, mortality and causes of death, severe morbidity, multiple births, congenital anomalies and readmission rates after discharge.

The principal sources of data for the CPSS perinatal health reports are Canadian vital statistics, hospitalization data (drawn from the Canadian Institute for Health Information [CIHI] Discharge Abstract Database, Manitoba Health's Perinatal Surveillance Database and the Canadian Congenital Anomalies Surveillance System), and the National Longitudinal Survey of Children and Youth (NLSCY). Population estimates and induced abortion statistics from Statistics Canada are also used.

## Results

### Maternal Mortality

Maternal mortality rates in Canada dropped from 8.2 per 100,000 live births in 1973–1977 to 4.4 per 100,000 live births in 1993–1997[1] and are among the lowest in the world. However, the World Health Organization (WHO) now routinely inflates reported maternal mortality ratios by a factor of 1.5 to take under-reporting into account. Canadian figures are no exception to this practice, so that the real rate of maternal death should be regarded as somewhat higher than the reported CPSS figures. Nevertheless, maternal death remains a rare event in Canada. The most common causes are, as in other industrialized countries, hypertensive disorders, pulmonary embolism, hemorrhage and ectopic pregnancy, all of which are conditions still amenable to further improvements in care.

### Maternal Morbidity

Because maternal deaths are rare, measures of severe maternal morbidity are more likely to indicate areas of concern with regard to health care services. In Canada, the most life-threatening conditions experienced by women during pregnancy, birth and the immediate postpartum period include amniotic fluid embolism, obstetric pulmonary embolism, eclampsia, septic shock, anaesthesia complications, cerebrovascular disorders, hemorrhage requiring either transfusion or hysterectomy, and rupture of the uterus. National figures regarding only one of these events, amniotic fluid embolism, are currently available[1] and indicate that while this event is rare (approximately 5.6 per 100,000 deliveries), it is associated with a high case fatality rate, approximately 80%. Amniotic fluid embolism accounts for 15% of all direct maternal deaths in Canada.

Other perinatal indicators that are available reflect far less life-threatening morbidity but are nevertheless of concern for women who experience them as well as for health care providers. Some considered here are maternal readmission after Caesarean section, operative deliveries by forceps or vacuum extraction, teenage pregnancy and induced abortion, births to older women, and breastfeeding rates.

### Maternal Readmission

Rates of maternal readmission after Caesarean birth have increased from 3.2 per 100 sections in 1990 to 3.9 in 1997; this requires explanation. In contrast, readmissions after normal vaginal deliveries have remained stable, at 2.5 per 100 deliveries over this period.[1] As the Caesarean section rate is also increasing (from 17.8 per 100 hospital deliveries in 1994 to 19.1 in 1997), there is an even greater potential for infant and maternal readmission to hospital in the future. These rates may be associated with shorter postpartum stays in hospital over the same period (vaginal deliveries: < 2 days for 3.2% of women in 1989 up to 25.6% in 1997; Caesarean sections: < 4 days for 2.1% of women in 1989 up to 31.3% in 1997[1]). At the very least, and in light of the increasing global and Canadian "popularity" of Caesarean birth, women need to be made aware of the increased risk of readmission of both themselves and their newborns after a caesarean section as compared with a normal vaginal delivery.

### Forceps and Vacuum Extraction Deliveries

Other perinatal indicators reveal areas of morbidity for mothers and infants. In 1997, wide variations occurred across the country in the rates of forceps deliveries (from < 3 per 100 vaginal deliveries in the Territories and Manitoba to over 8 per 100 in New Brunswick, Newfoundland and Labrador, Nova Scotia and Ontario) and vacuum extractions (from about 4–6 per 100 vaginal deliveries in hospital in the Northwest Territories, Nova Scotia and Prince Edward island to 14–15 per 100 deliveries in Saskatchewan and the Yukon), indicating local treatment preferences.

### Teenage Pregnancy and Induced Abortion

Further indicators of concern relate to teenage pregnancy and induced abortions. The live birth rate among teens aged 15 to 19 has declined in Canada from 25.8 per 1,000 in 1981 to 19.9 in 1997. Similarly, the birth rate among females aged 10 to 14 has declined, from 0.29 per 1,000 in 1981 to 0.22 in 1997.[1] In 1997, 5.6% of all births in Canada were to women between 15 and 19 years of age, whereas girls under 15 contributed less than an additional 1%.

In 1997, 21.5 per 1,000 women between 15 and 19 had induced abortions, as did 2.6 per 1,000 under 15 years. In contrast, 33.9 per 1,000 women between the ages of 20 and 24 had an induced abortion. The total induced abortion rate for Canada in 1997 was 16.8 per 1,000 women, an increase from 14.6 in 1990.[2] In 1995, abortions for women under 20 years reflected approximately 20% of all abortions performed in Canada.[2] Induced abortion figures are likely to be significantly under-reported, however, as medically/pharmacologically induced abortions performed in physicians' offices are not included in these figures. Surgical abortions that are performed by physicians in their offices are also not recorded when the office is not designated as an abortion facility service. The figures should, therefore, be interpreted with caution, particularly because medical methods of pregnancy termination are likely to be used with increasing frequency in future. Nevertheless, even allowing for under-reporting, it appears that the rates of teenage pregnancy in Canada are declining while abortion rates are increasing. It is evident that there is still room for much improvement regarding the prevention of unwanted pregnancies, particularly in teenagers.

### Births to Older Mothers

Births to older mothers, on the other hand, are an issue of increasing concern as these carry higher risk for both mother and infant. The proportion of births to older women has been increasing steadily over the past two decades. In 1997, 30.2% of all live births in Canada were to women aged 30 to 34 years, 12.4% to women aged 35 to 39 and 1.9% to women over 40. Older women are more susceptible to complications of pregnancy, such as spontaneous abortion, gestational diabetes, hypertension, pre-eclampsia, and placenta previa. Common labour complications for older mothers include malpresentation, cephalopelvic disproportion, protracted labour, operative deliveries and postpartum hemorrhage. For example, in 2000 the preterm birth rate among women over 34 years was the highest of all age groups: 8.8 per 1,000 births as compared with the national average preterm birth rate of 7.5 in the same year.[3]

### Breast-feeding Rates

Breast-feeding rates are, at first glance, reasonable but do not stand up well to close scrutiny. In 1997, 73% of mothers initiated breast-feeding, although rates as measured by the NLSCY dropped to 33% at two months after birth,[4] suggesting that postpartum support for breast-feeding could be considerably strengthened. At present, there are no clear national figures on the rates or duration of exclusive breast-feeding in Canada. Both the Breastfeeding Committee for Canada and the CPSS have expressed concerns about this. The CPSS has instituted steps to obtain such measures in future.

### Infant Mortality in Canada

Infant mortality ranged from 5.3 to 8.8 per 1,000 live births between 1990 and 2000.[1,3] These indicators are among the lowest in the world. There are, however, unacceptable disparities between subpopulations within the country on many infant health indicators, including the key one of infant deaths. For instance, despite universal health insurance, socio-economic status influences health outcomes such that low income groups showed a 1.6 times greater risk of infant deaths than high income groups in 1991. Perinatal health among First Nations, Métis and Inuit populations also needs special attention. The rates of stillbirth and perinatal mortality among registered Indians have been estimated to be about double the Canadian average, and rates among the Inuit in the Northwest Territories are about two and a half times the rates for Canada as a whole.[1] Addressing these health disparities remains a challenge within Canada and reflects a still current need to promote equitable health care across social and cultural disparities.

On the other hand, some perinatal indicators show decided improvements with regard to infant mortality in recent years. In particular, the incidence of infant mortality due to congenital malformations has declined significantly, from 3.1 per 1,000 live births in 1985 to 1.9 in 1995. These figures likely reflect increased prenatal diagnosis and termination of affected pregnancies together with improvements in care of infants with congenital anomalies.

### Infant Morbidity

While maternal and infant mortality are primary perinatal care outcome measures, these expose only the tip of the iceberg of perinatal health care concerns. Measures of morbidity rather than mortality are of more importance when mortality rates are low, as in Canada. Key indicators of infant morbidity are preterm and multiple birth rates.

#### Preterm Births

One of the most significant variables indicative of infant morbidity is the preterm birth rate. Rates of preterm birth in Canada increased rather than decreased between 1981 and 2000 (from 6.4 per 100 deliveries to 7.5),[1,3] probably because of increases in multiple births and obstetric interventions such as the increasing use of ultrasound. Concurrently, the rate of stillbirths declined, suggesting that increasing use of ultrasound saves the lives of some babies, albeit at the risk of preterm delivery.

#### Multiple Births

The multiple birth rate has been increasing steadily for some decades but has accelerated rapidly more recently. This increase is of considerable concern, not only because multiple births carry higher risks but also because they are associated with preterm births. The multiple birth rate has increased from 18.2 per 1,000 total births in 1974 to 19.3 in 1980, 20.8 in 1990 and 25.0 in 1997. This increase is paralleled in other industrialized countries and is associated with increases in the proportion of older women giving birth and with increasing use of infertility treatments. It is not clear how much of the multiple birth rate in Canada can be attributed to infertility treatments, but in the United States in 1996 (the last year reported), 38.9% of all births associated with in vitro fertilization and assisted reproductive technologies were multiple gestation deliveries.[5] The rate of multiple births among these mothers is rising by about 3% per annum.[6]

Although such technological advancement is welcomed, there must also be a recognition that multiple births (which frequently follow infertility treatments) are associated with higher rates of fetal and infant mortality and morbidity. In the United Kingdom, the perinatal mortality rate is reported as 1.4% among singletons, 5.8% among twins and 9.1% among triplets.[7] In Canada, serious morbidity is higher among twins and triplets: for example, cerebral palsy rates among twins and triplets is estimated to be 47 times and 8 times higher respectively than among singletons.[1]

The second concern regarding multiple births is their associated increased risk of preterm birth. Preterm births have increased as a proportion of multiple births from 33% in 1974 to 40% in 1981–1983, 50% in 1992–1994 and 53.5% in 1997. In 2000, 51.5% of twin pregnancies resulted in preterm births and 97.7% of triplet or higher multiple births did so.[3] These increases in preterm births are dramatic compared with the more modest rates of preterm births among singletons (6.2 per 100 births).[3] Notwithstanding these figures, the overall rate of preterm birth in Canada remains fairly low, at 7.5 per 100 in 2000.[3]

## Discussion

### Data Gaps

While the quality of data sources is generally good, there are still challenges that need to be overcome to ensure uniformly high quality data. These include obtaining data from across the country in a more timely fashion, ensuring completeness of all data, tracking more obscure or complex diagnoses, reporting events that occur outside hospitals such as home births and spontaneous abortions, and ensuring uniformity in defining, recording and reporting perinatal events across provinces and territories. Significant attempts are constantly being made by the CPSS to overcome the remaining obstacles to high-quality coverage of perinatal indicators. These are discussed in more detail in the CPSS perinatal health reports.

One recommendation for strengthening the current system of perinatal surveillance in Canada through the CPSS concerns the monitoring of economic implications of perinatal care. At present there is no systematic review of the costs of specific perinatal practices in Canada. Health care is expensive for the country, and some aspects, such as neonatal intensive care, are extremely costly. Multiple births, with their higher risk of morbidity, mortality and preterm birth, are associated with greater use of neonatal intensive care units, longer hospital stays, and higher rates of medication. Later childhood problems may be the most serious, although these are difficult to quantify. With regard to costs, low birth-weight babies without disability require two times, and those with disability five times, as much health and education expenditure to age 9 as singleton babies of normal weight.[8] The implications of these demands on the family, the growing child, the educational system and the welfare system may all be substantial. The physiological, psychological and economic costs may be severe.

Given that some particularly expensive perinatal care practices, such as infertility treatments (which result in increased use of neonatal intensive care units) or surgical delivery, appear to be on the increase in Canada, the importance of obtaining economic indicators of these practices is growing – not to restrict the use of expensive but necessary care, but rather to evaluate the unnecessary or excessive use of practices that might, in addition to being non-evidence-based, also be costly. The CPSS is aware of this concern and is planning to consider assessment of economic indicators in future.

## Recommendations

• Economic indicators should be included as part of CPSS surveillance of perinatal health.

• CPSS should provide an evaluation of how well Canada matches up to international standards of perinatal care (currently on the agenda of the CPSS Steering Committee).

• WHO has developed recommendations for perinatal practices that are applicable globally. The CPSS should establish a means of comparing Canada's practices with WHO standards (currently on the agenda of the CPSS Steering Committee).

## Notes

The views expressed in this report do not necessarily represent the views of the Canadian Population Health Initiative, the Canadian Institute for Health Information or Health Canada.
